# The New Era of Organ Transplantation in Greece: Time to Converge With the Western World

**DOI:** 10.3389/ti.2025.14668

**Published:** 2025-06-12

**Authors:** Dimitrios Moris, Emmanouil Giorgakis

**Affiliations:** ^1^ Department of Surgery, MedStar Georgetown Transplant Institute, Washington, DC, United States; ^2^ Division of Abdominal Transplantation, Department of Surgery, University of North Carolina at Chapel Hill School of Medicine, Chapel Hill, NC, United States

**Keywords:** organ allocation, policies, organ and tissue donation and transplantation, Greece, innovation

Dear Editors,

Greece has a relatively long history in organ transplantation with the first successful kidney transplant being performed in 1968. Over the last decade, in the midst of the worst financial crisis in the history of the country [1], due to the brain drain and the lack of political and administrative vision, the organ donation and transplantation reached a historic low [2, 3]. Over the recent few years, a reversal of the trend is noticed. The current momentum, the rising donor rates, the establishment of an Academic Department for Liver Transplantation in Athens, the Pancreas Transplant Unit in the University Hospital of Patras and the Onassis National Transplant Center, structural reforms of the Hellenic Transplant Organization (HTO; including placement of local transplant coordinators in Intensive Care Units) and the brain regain, might facilitate the effort that Hellenic Transplant System is making to converge with the international transplant standards.

Compared to our analysis from almost 10 years ago [2], there are some changes in the number and structure of the transplant programs. Greece has 5 kidney transplant programs (with 1 program that has extensive experience of >4,000 deceased-donor and 1,000 living-donor kidney transplants) and another 1 new kidney transplant program expected to be fully operational in the next few years. Currently, Greece has 2 active liver transplant programs performing deceased-donor liver transplants, based in Thessaloniki and Athens. The latter recently successfully performed the first couple living-donor liver transplants, with the assistance of foreign collaborators. Two of the kidney programs are licensed to perform pediatric kidney transplants. Moreover, now there are 2 pancreas transplant programs established, and 1 cardiac and lung transplant program. Currently, there is no active small bowel or dedicated pediatric transplant program. As mentioned before [2], children older than 14 years are registered and get priority on the adult kidney list, whereas children younger than 14 years are still referred to centers abroad. All transplant programs are public, and no transplantation license has been issued in the private health sector.

In a closer look of the transplant frame in Greece, a “soft opt-out” consent system has been in effect since 2023 (an opt-in system was in place since 2017) and there has been clear transplant legislation that defines brain death. Ongoing convergence of our legislature to the international guidelines on the role of ancillary tests to confirm brain death, could mitigate the high levels of public distrust in the system. Pediatric live donation is prohibited. There are some provisions for donation after circulatory death since 2023 but unfortunately no DCD organs have been transplanted to date as well as no clear guidelines regarding donor warm ischemia time have been defined. Moreover, no provisions exist regarding directed or non-directed altruistic donation: all policies that, if implemented, will definitely increase the pool of available organs [4].

A recent workforce analysis that pioneered the use of conceptual frameworks as resources to guide the evaluation and the transformation of organ donation and transplantation in Greece [5], showed that medical staffing levels in Hellenic Transplant System were below the average of other European and Mediterranean countries across all specialties (including surgeons, physicians, anesthesiologists, and others) [6]. There is also an urgent need to expand operating room capacity for both donation (national donor center) [7] and transplantation procedures, and to improve access quality of ancillary services (radiology, transplant infectious diseases, endoscopy, interventional radiology, pathology and histocompatibility testing etc.) across all existing units.

Using data from IRODaT (International Registry on Organ Donation and Transplantation), since 2019 (time of financial recovery of Greek Economy after 10 years of financial regression), there was a gradual increase in actual donation from deceased donors per year, from 5.5 deceased donors/million population and 6.2 living donors/million population in 2019 to 18.2 and 12.1 in 2024 respectively (Figure 1A), which is higher than the previously reported period in our analysis 10 years ago (average 4 and 5, respectively) [2]. However, from the 188 deceased donors performed, organs from only 102 donors were utilized in 2024. The number of utilized donors during the same period showed similar trajectory with the actual donors (data are not shown), however the IRODAT data are not granular enough to provide more insights about the reasons for discarding organs, that is a known phenomenon observed in many countries [8]. Moreover, there was a gradual increase in the number of solid organ transplants during the same period (2019–2024), with 178 kidney, 33 liver and 15 heart, 0 lung and 0 pancreas transplants in 2019 to 277 kidney, 55 liver, 24 heart and 12 lung and 2 pancreas transplants in 2024. Three multiorgan transplants (2 kidney-pancreas and 1 liver-kidney) were performed. No intestinal transplants or transplants from DCD were performed during the same period (Figure 1B) [9].

**FIGURE 1:**
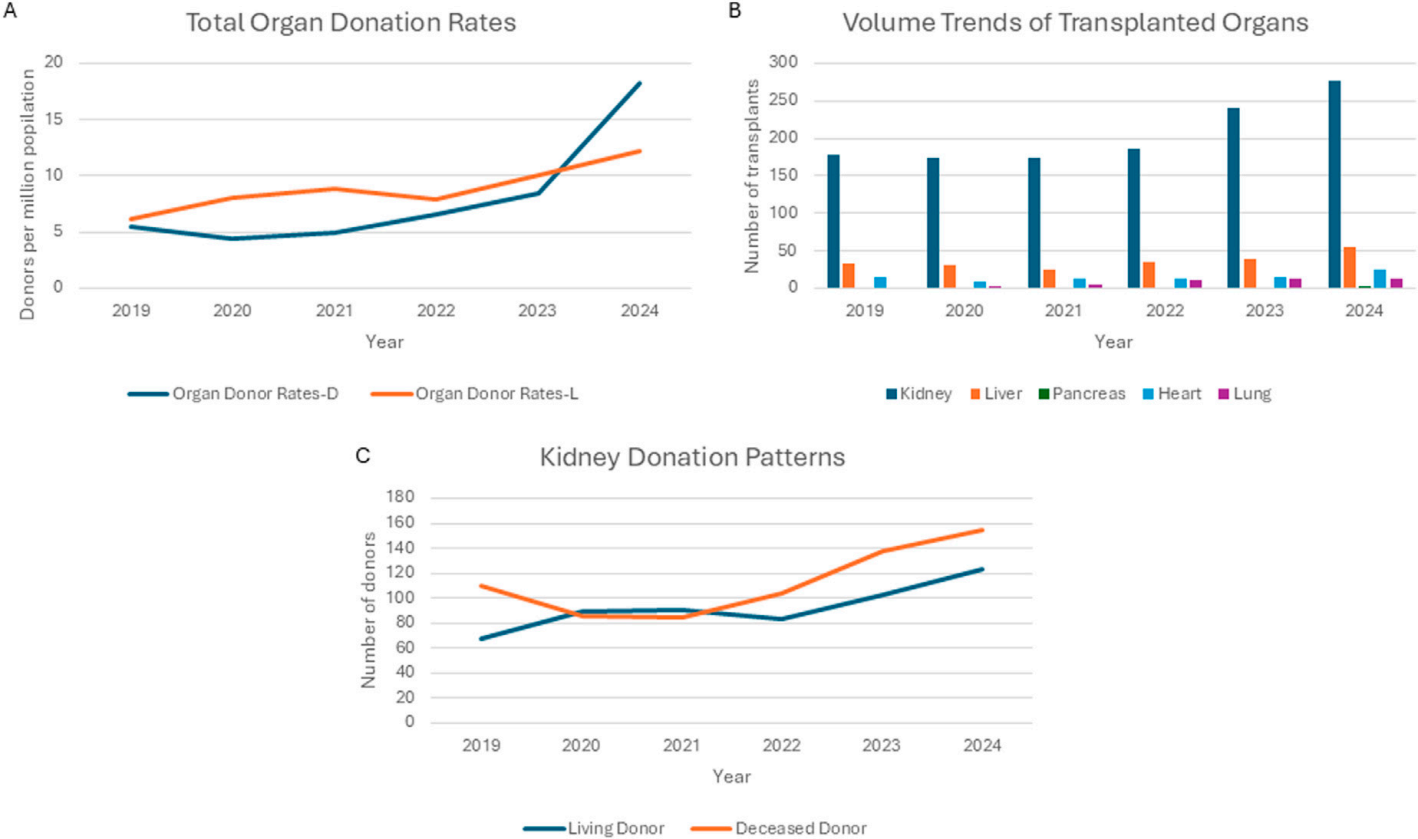
**(A)** Trends of total organ donation rates during the period 2019–2024. **(B)** Volume trends of transplanted organs during the period 2019-2024. **(C)**. Increasing kidney donation numbers during the same period (orange line: deceased donors, blue line: living donors).

It is clear from the data presented above that the performance of the Hellenic Transplant System has improved during the recent years, especially if we focus on the years after the financial regression. This improvement is multifactorial. First, Greece is undergoing a period of political, governmental and administrative stability, that allowed better mid- and long-term planning as well as sustainability of the transplant system. Of course, the Hellenic Transplant System requires better organization and deep structural reforms, such as establishment of national or regional transplant center(s); better staffing, including specialized coordinators; national donor and recipient registries; transparency at the level of individual programs as well as nationwide in outcome reporting, quality maintenance and improvement initiatives. The current political environment appears supportive to the changes needed. Moreover, the financial recovery that Greek economy had the last few years allowed for more incentives to be offered to all parties involved (facilities and staff), to be compensated for their work in organ donation and transplantation. Furthermore, the successful implementation of minimally invasive techniques for kidney donation (with the establishment of a high volume center that contributes to the rising living donor numbers found in our analysis, Figure 1C) [10] the successful performance of cases of adult living liver donation [11] as well as the simultaneous transplantation of renal and pancreatic allografts in patients with end-stage renal disease and diabetes, really boosted public trust around transplantation personnel and their mission, and acted as a great national campaign strategy for organ donation.

Unfortunately, the Hellenic Transplant System made no progress in improving the leadership capacity and the workforce to support a modern transplant system according to international standards. It is obvious that living donation in kidney transplantation can be a sustainable solution to organ demand. However, in liver transplantation, living donation is more nuanced and requires significant expertise on patient selection, and transparency on the listing process (low vs. high MELD patients; exemption points for transplant oncology patients; streamlined process to list patients with high MELD; split or reduced grafts for adult and pediatric transplant recipients). Also, the adoption of DCD, and at later staged the inclusion of normothermic regional perfusion (NRP) as well as the use of machine perfusion as a primary organ storage modality will allow the utilization of extended criteria donors and augment the available organ pool [12, 13]. Especially, in a country like Greece, given its topography and the numerous islands, machine perfusion could allow utilization of organs with longer cold ischemia times without compromising outcomes.

Despite the brain regain that is currently taking place, the academic surgical and transplant community remain resistant to change. There is a plethora of Greek surgeon-scientists who had left the country during the years of financial crisis and currently possess the expertise and the zest to join the effort of Hellenic Transplant System modernization. Greek transplant physicians and surgeons currently practicing abroad could cover the obvious gaps in technical skills (in living liver donation, laparoscopic/robotic living donation/transplant, pancreas and bowel transplant, multiorgan transplant, extended criteria donor utilization, advanced organ procurement and machine perfusion modalities, surgical innovation), scientific extroversion and networking (through international collaborations and research initiatives), patient care optimization (e.g., through modernization of immunosuppressive protocols, personalized care, transplant oncology, donor and recipient evaluation, inpatient and outpatient care), transplant logistics, and training of the next-generation of transplant professionals.

Transplantation is considered the epitome of surgical technique. It is philosophically based on the pillars of altruism (organ donation), and utilitarianism (equitable distribution of life-saving organs, prioritizing those most in need). However, transplantation is a delicate system to thrive. Transplant networks remain healthcare’s proverbial canary in the mine: it takes a robust healthcare system and an empathetic, altruistic society for transplantation to be operational and thrive; and *vice versa*, transplantation as an organism will suffer if a healthcare system is under strain or if a socioeconomical system is in decline, as it has been shown in our recent past.

Until recently, Greece lacked the vision to invest on comprehensive organ donation and transplantation services, the benefit of which is certainly indisputable. The current comparative socioeconomical and political stability has afforded an opportunity for transplantation to reach its long-awaited puberty in Greece; yet it is still fledgling, and the growth spurt should be steep if it is to catch up with the transplant revolution witnessed elsewhere. It must therefore be nurtured by the political and healthcare stakeholders, who, among other, should embrace a more meritocratic approach in attracting and retaining talent ostracized during the country’s lean years, currently pushing transplant boundaries overseas.

The recent steps forward, in terms of social acceptance, innovation and outcomes should be the steppingstone to the foundation of a modern transplant system that Greek medical tradition and patient population deserve.

## Data Availability

The raw data supporting the conclusions of this article will be made available by the authors, without undue reservation.

## References

[1] Moris D Zavos G Menoudakou G Karampinis A Boletis J. Organ Organ donation during the financial crisis in Greeceonation during the Financial Crisis in Greece. Lancet (2016) 387(10027):1511–-2. 10.1016/S0140-6736(16)30130-1 27115977

[2] Moris D Menoudakou G Zavos G. Organ Transplantation in Greece. Transplantation (2016) 100(8):1589–91. 10.1097/TP.0000000000001349 27454912

[3] Giorgakis E Singer AL Khorsandi SE Prachalias A. Transplantation Transplantation Crisis at the Time of Economic Recession in Greecerisis at the Time of Economic Recession in Greece. Public Health (2018) 160:125–-8. 10.1016/j.puhe.2018.03.031 29803187

[4] Moris D Tsilimigras DI Bokos J Vernadakis S. Organ Donation Organ Donation After Circulatory Death in Greece: Time to Considerfter Circulatory Death in Greece: Time to Consider. Exp Clin Transpl (2020) 18(4):539–40. 10.6002/ect.2019.0100 31424355

[5] Johnston-Webber C Mah J Streit S Prionas A Wharton G Mossialos E A Conceptual Framework for Evaluating National Organ Donation and Transplantation Programs. Transpl Int (2023) 36:11006. 10.3389/ti.2023.11006 37334013 PMC10273098

[6] Johnston-Webber C Prionas A Wharton G Streit S Mah J Boletis I The National Organ Donation and Transplantation Program in Greece: Gap Analysis and Recommendations for Change. Transpl Int (2023) 36:11013. 10.3389/ti.2023.11013 37305340 PMC10249496

[7] Johnston-Webber C Wharton G Mossialos E Papalois V. Maximising Potential in Organ Donation and Transplantation: Transferrable Paradigms. Transpl Int (2022) 35:11005. 10.3389/ti.2022.11005 37304736 PMC10249497

[8] Moris D Schmitz R Dimitrokallis N Schmidt T Vernadakis S. The Paradox of Increasing Waiting List Mortality and Declining Utilization of Deceased Donor Grafts in Kidney Transplant. Exp Clin Transpl (2021) 19(1):92–3. 10.6002/ect.2019.0427 32490764

[9] DTL. Database (2023). Available online at: https://www.irodat.org/?p=database&c=GR&year=2023#data (Accessed May 21, 2025).

[10] Vernadakis S Marinaki S Darema M Soukouli I Michelakis IE Beletsioti C The Evolution of Living Donor Nephrectomy Program at A Hellenic Transplant Center. Laparoscopic vs. Open Donor Nephrectomy: Single-Center Experience. J Clin Med (2021) 10(6):1195. 10.3390/jcm10061195 33809339 PMC8001196

[11] Machairas NS GC. Living Donor Liver Transplantation Could Solve Shortage of Liver Grafts in Greece. Hell J Surg (2024) 94(3):165–7.

[12] Tingle SJ Dobbins JJ Thompson ER Figueiredo RS Mahendran B Pandanaboyana S Machine Machine Perfusion in Liver Transplantationerfusion in Liver Transplantation. Cochrane Database Syst Rev (2023) 9(9):CD014685. 10.1002/14651858.CD014685.pub2 37698189 PMC10496129

[13] Malinoski D Saunders C Swain S Groat T Wood PR Reese J Hypothermia or Machine Perfusion in Kidney Donors. N Engl J Med (2023) 388(5):418–26. 10.1056/NEJMoa2118265 36724328

